# Supplementary material for: The genome sequence of a tachinid fly, Tachina lurida (Fabricius, 1781)
Source: Wellcome Open Res. 2023 Jul 7;8:288. [Version 1] doi: 10.12688/wellcomeopenres.19635.1 (PMC10448141; doi:10.12688/wellcomeopenres.19635.1)
Supplement: Peer review history

# Peer review: Reviewer response for version 1

**DOI:** 10.21956/wellcomeopenres.21751.r65719

**Published:** 2023-08-23

**Authors:** Jerome H L Hui

**Affiliations:** 1 Chinese University of Hong Kong, Hong Kong, China

Falk and colleagues report the genome sequence of tachinid fly *Tachina lurida *(Fabricius, 1781). This species can be found in Britain according to past records, such as NatureSpot, iNaturalist and NBNatlas Scotland. Molecular data of this species are scarce prior to this report, and are mainly confined to COI sequences deposited to the NCBI database. Therefore, this new genome resource will be useful for further studies, ranging from revealing their population structures, dissecting host plant-insect relationships, to understanding their evolution with other insects.

 This genome resource is excellent from the summary statistics, with high BUSCO numbers, high sequence continuity, and majority of sequences contained on the 6 pseudochromosomes (plus mitochondrion). All in all, this is a valuable contribution.

Are sufficient details of methods and materials provided to allow replication by others?

Yes

Is the rationale for creating the dataset(s) clearly described?

Yes

Are the datasets clearly presented in a useable and accessible format?

Yes

Are the protocols appropriate and is the work technically sound?

Yes

Reviewer Expertise:

Genomics, evolution, invertebrates

I confirm that I have read this submission and believe that I have an appropriate level of expertise to confirm that it is of an acceptable scientific standard.

# Peer review: Reviewer response for version 1

**DOI:** 10.21956/wellcomeopenres.21751.r64459

**Published:** 2023-08-07

**Authors:** Atsushi Sugie

**Affiliations:** 1 Niigata University, Niigata, Japan

The methodologies, techniques, and protocols employed in this study for genome sequencing and transcriptome sequencing (RNA sequencing) do not exhibit any significant issues or suspicious aspects. Each step corresponds to a specific objective and each procedure is appropriately designed. Thus, this paper is well-prepared for indexing, presenting the identified genome sequence of *Tachina lurida*.

Are sufficient details of methods and materials provided to allow replication by others?

Yes

Is the rationale for creating the dataset(s) clearly described?

Yes

Are the datasets clearly presented in a useable and accessible format?

Yes

Are the protocols appropriate and is the work technically sound?

Yes

Reviewer Expertise:

Molecular biology, neurobiology, genetics

I confirm that I have read this submission and believe that I have an appropriate level of expertise to confirm that it is of an acceptable scientific standard.
